# Three-Dimensional Magnetic Resonance Imaging Reveals the Relationship Between the Control of Vertigo and Decreases in Endolymphatic Hydrops After Endolymphatic Sac Drainage With Steroids for Meniere's Disease

**DOI:** 10.3389/fneur.2019.00046

**Published:** 2019-02-04

**Authors:** Taeko Ito, Hiroshi Inui, Toshiteru Miyasaka, Tomoyuki Shiozaki, Shohei Matsuyama, Toshiaki Yamanaka, Kimihiko Kichikawa, Noriaki Takeda, Tadashi Kitahara

**Affiliations:** ^1^Department of Otolaryngology- Head and Neck Surgery, Nara Medical University, Kashihara, Japan; ^2^Inui ENT Clinic, Sakurai, Japan; ^3^Department of Radiology, Nara Medical University, Kashihara, Japan; ^4^Department of Otolaryngology, University of Tokushima School of Medicine, Tokushima, Japan

**Keywords:** endolymphatic hydrops, Meniere's disease, surgery, MRI, endolymphatic sac

## Abstract

Meniere's disease is a common disease, that presents with recurrent vertigo and cochlear symptoms. The pathology of Meniere's disease was first reported to involve endolymphatic hydrops in 1938. The endolymphatic sac is thought to have a role to keep the hydrostatic pressure and endolymph homeostasis for the inner ear. As a surgery for intractable Meniere's disease, endolymphatic sac drainage with intraendolymphatic sac application of large doses of steroids is performed to control the endolymphatic hydrops and preserve or improve inner ear function. In the present study, to observe the effect of this surgery, we calculated the endolymphatic space size using 3-Tesla magnetic resonance imaging (MRI) 4 h after intravenous injection of gadolinium enhancement at two time points: just before surgery and 2 years after. To reveal the condition of the endolymphatic space, we constructed three-dimensional MR images semi-automatically and fused the three-dimensional images of the total fluid space of inner ear and the endolymphatic space. After fusing the images, we calculated the volume of the total fluid space and endolymphatic space. Two years after surgery, 16 of 20 patients (80.0%) showed relief from vertigo/dizziness and reductions in the ratio of the volume of the endolymphatic size to the total fluid space of inner ear. Endolymphatic sac drainage with intraendolymphatic sac application of large doses of steroids could control vertigo/dizziness and decrease the endolymphatic hydrops. These results indicate that endolymphatic sac drainage is a good treatment option for patients with intractable Meniere's disease. In addition, volumetric measurement of inner ear volume could be useful for confirming the effect of treatments on Meniere's disease.

## Introduction

Meniere's disease (MD) is a chronic disease that presents with recurrent vertigo, tinnitus, aural fullness, and fluctuating hearing loss. Its pathology was first reported in 1938 to be endolymphatic hydrops (EH) through two well-known temporal bone studies (1, 2). Recently, some genes involved in immune responses have been associated with clinical phenotypes of MD, MICA, and TLR10 are associated with hearing loss progression in patients with MD and the common variant rs11096955 in the TLR10 gene has been linked to the progression of hearing loss in patients with bilateral MD (3). Li et al. used a molecular network-based method using a random walk with restart algorithm to predict genes potentially involved in MD (4) and found two common allelic variants in the NFKB1 gene (rs3774937 and rs4648011) to be associated with hearing loss progression in patients with MD. However, the mechanisms underlying EH generation (i.e., the relationship between stress and inner ear water metabolism) have remained unclear. In addition, detection of EH in patients with MD clinically using morphological examinations is considered to be difficult. In 2007, Nakashima et al. reported that 3-Tesla magnetic resonance imaging (MRI) with gadolinium (Gd) enhancement could visualize the EH in patients with MD (5). In 2016, we reported a method to evaluate the endolymphatic space (ELS) size or EH with volumetric measurement on three-dimensional MRI (6). A combination of these two methods can allow objective and quantitative evaluation of the EH.

When conservative medical treatments for MD fail, surgical strategies are usually performed according to the treatment guidelines for MD (7). The first surgical procedure of endolymphatic drainage (ELSD) for MD was described by Portmann in 1927, who opened the endolymphatic sac in order to reduce the endolymphatic pressure (8). ELSD is still commonly performed and yields complete vertigo control in 42–88% of patients (9). MD is thought to be caused by immune, metabolic, infectious, traumatic, or other insults to the inner ear associated with a temporarily dysfunctional endolymphatic sac (10, 11). Among these conditions, immune-mediated responses in the inner ear endo-organs such as the endolymphatic sac, stria vascularis, and spiral ligament are thought to be the main reasons for the progression of MD (3, 12). Thus, systemic administration and/or local perfusion of corticosteroids into the middle ear, an immunosuppressive or anti-inflammatory therapy, are used for the strategies for patients with intractable MD (13). In 2008, Kitahara et al. reported a new therapeutic strategy that combined ELSD with intraendolymphatic sac application of steroids for intractable MD, which controlled vertigo/dizziness quite well (14). ELSD with/without intraendolymphatic sac application of steroids is reported to be useful to control vertigo/dizziness, but there is no paper that objectively and quantitatively describes the condition of the ELS or EH after ELSD.

In the present study, we performed volumetric measurement of ELS at two time points—just before and 2 years after ELSD—to objectively and quantitatively evaluate the condition of EH and the effect of ELSD.

## Materials and Methods

The Medical Ethics Committee of Nara Medical University approved this study (certificate number: 0889). All subjects gave written informed consent in accordance with the Declaration of Helsinki.

### Patients

Twenty patients with a clinical diagnosis of MD according to the 1995 American Academy of Otolaryngology–Head and Neck Surgery (AAO-HNS) guidelines (7) and Bárány Society criteria (15) were enrolled in the present study. The patients had intractable MD, which was defined as the patients with recurrent vertigo/dizziness for at least 6 months and whom systematic medical treatments and psychological management had been applied and had failed. They received ELSD at our university hospital from February 2014 to July 2016.

### Surgical Treatment

Kitahara et al. have reported the procedure used for ELSD with steroids (14). Briefly, simple mastoidectomy was performed to clearly expose the endolymphatic sac, which was opened with an L-shaped incision and filled with 20 mg of prednisolone (Predonine; Shionogi, Japan). Then, a bundle of absorbable gelatin film (Gelfilm; Pfizer, Japan) was inserted into the sac and small pieces of absorbable gelatin sponge soaked in a high concentration (3.3 mg/ml) of dexamethasone (Decadron, Aspen Japan, Japan) were placed inside and outside the sac.

### Functional Examinations

Definitive vertigo/dizziness lasting more than 20 min was regarded as a vertigo attack according to the modified 1995 AAO-HNS guidelines (7). The frequency of vertigo before ELSD (“before”) was calculated based on the number of vertigo attacks during the 6 months before ELSD. Frequency after ELSD (“after”) was calculated based on the number of vertigo attacks during the 6-month period between 18 and 24 months after ELSD.

### Functional Measurement

#### Hearing Test

Hearing function was measured by a pure-tone audiometer (AA-78; Rion, Japan) and was evaluated based on the four-tone average formulated by (a + b + c + d)/4 (a, b, c, d are hearing levels at 0.5, 1.0, 2.0, and 4.0 kHz, respectively) according to the modified 1995 AAO-HNS guidelines (7, 16). The worst hearing level during the 6 months before ELSD was defined as the hearing level before ELSD (“before”). The worst hearing level during the 6 months between 18 and 24 months after ELSD was used for evaluation of the hearing level at 2 years after ELSD (“after”). Differences in hearing levels >10 dB before and after ELSD were regarded as “better.” Differences smaller than −10 dB were regarded as “worse” and values in between better and worse were considered to indicate “no change.”

#### Glycerol Test

The glycerol test (G-test) was performed to detect EH once before ELSD (“before”) and once between 18 and 24 months after ELSD (“after”). Hearing function was measured just before oral administration of glycerol (1.3 mg/kg) and 3 h later. This test is considered positive in pure-tone audiometry if there is a 10-dB improvement at two or more frequencies between 0.25 and 2.0 kHz.

#### Electrocochleography

We also performed electrocochleography (EcoG) to detect EH at the same time as the G-test (“before” and “after”). The silver-ball electrode for EcoG (COC-1; unique medical, Japan) was placed on the upper wall of the deepest part of the ear canal near the eardrum, and measurements were obtained with click sounds using NeuropackX1 (Nihonkoden, Japan). This test is considered positive if –SP/AP > 0.37 (the average + 2 × standard deviation of healthy subjects) at our hospital.

### MRI Assessments

We performed MRI assessments just before ELSD (“before”) and 2 years after ELSD (“after”).

#### Pre-processing

Naganawa et al. (17) described using MRI 4 h after intravenous injection of Gd as a tool for imaging EH. Briefly, in the present study, MRI measurements were performed 4 h after intravenous administration of a single dose (0.2 ml/kg or 0.1 mmol/ kg body weight) of Gd-diethylenetriaminepentaacetic acid dimethylamide (Magnescope; Guerbet, Tokyo, Japan). A 3-Tesla MRI unit (MAGNETOM Verio; Siemens, Erlangen, Germany) with a 32-channel array head coil was adopted in the present study. Special sequences for revealing the endolymphatic and perilymphatic fluid were used, as proposed by Naganawa et al. (17).

All patients underwent the following protocols: heavily T2-weighted (hT2W) MR cisternography (MRC) for an anatomical total lymph fluid reference; hT2W 3-D fluid-attenuated inversion recovery sequences with an inversion time of 2,250 ms, yielding positive perilymph images (PPI); and hT2W 3-D inversion recovery with an inversion time of 2,050 ms, yielding positive endolymph images (PEI). After image acquisition, we obtained a hybrid image of the reversed image of the positive endolymph signal and the negative image of the positive perilymph signal after motion correction by subtracting PEI from PPI, as proposed by Naganawa et al. (17, 18). In this protocol, pixels with a negative value were regarded as the ELS or EH.

Detailed scan parameters for hT2W MRC were as follows: variable refocus flip angle 3-D-turbo spin-echo (sampling perfection with application-optimized contrasts using different flip angle evolutions [SPACE]); repetition time (TR), 4,400 ms; echo time (TE), 544 ms; constant flip angle mode; matrix size, 322 × 384; slices per slab, 104; slice thickness, 1.0 mm; field of view, 150.9 × 180.0 mm; and bandwidth, 434 Hz/pixel.

**Figure 1:**
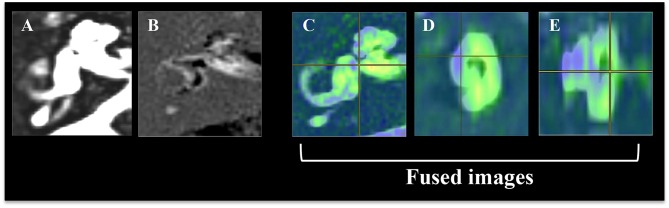
Reconstruction procedure for color rendering and fusion of 3-D images of the inner ear fluid space and 3-D image of the endolymphatic space (ELS). **(A)** Image of the total inner ear fluid space. **(B)** Image of the endolymphatic space. **(C)** Fusion images, axial view of the inner ear. **(D)** Fusion images, sagittal view of the inner ear. **(E)** Fusion images, coronal view of the inner ear. The image of the total inner ear fluid space obtained by the T2-SPACE sequence was fused with the image of endolymphatic space by PPI-PEI.

**Figure 2:**
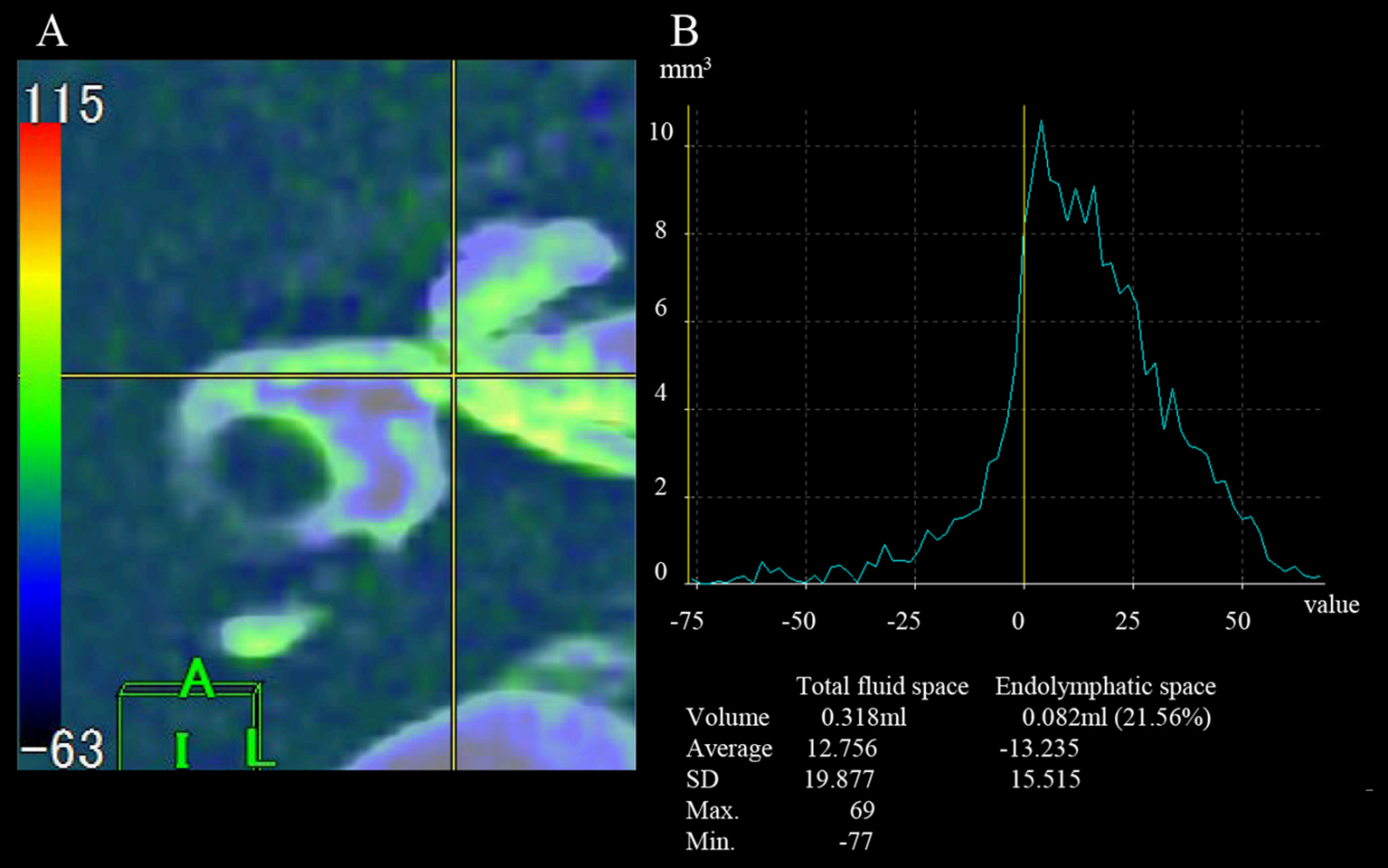
Measurement of the volume of total inner ear fluid space and the endolymphatic space. **(A)** Axial field of view for the fusion image. **(B)** A graph for the measurement of volume. The gray area on the color bar indicates a value of 0 on the graph (yellow line). A negative value indicates the volume of the endolymphatic space.

The area of ELS was first recognized on our workstation (Virtual Place; AZE, Ltd., Tokyo, Japan) (Figure 1). Because ELS voxels have negative signal values and the perilymph space voxels have positive signal values, PPI and PEI were transferred, and PEI images were subtracted from the PPI images using the fusion program included with the software in our workstation. The borderline between the gray area and the green area of the color bar in our subtracted image was considered as zero-value in our program (Figure 2). Additionally, the subtracted image was consistent with the “PPI-PEI” image. In this process, areas that had lower intensity than gray were recognized as the ELS areas.

#### Method of Fusing Images

As we reported in 2016 (6, 19), source images of the inner ear fluid space (SPACE sequence image) and ELS (“PPI-PEI” image) were reconstructed on our workstation. The inner ear fluid space was manually identified from the surrounding structures by the object extraction function and cut tool of the workstation. A high-quality 3-D image was constructed semi-automatically using both anatomical and tissue information by fusing the 3-D images of the inner ear fluid space and 3-D ELS.

#### MR Imaging Evaluation

##### Conventional two-dimensional evaluation

Two neurotologists, who were blinded to the clinical progress of the patients, evaluated the MRI findings. If their evaluations differed, a third neurotologist made the final decision. For evaluation of EH, the area of total fluid space in the cochlea and vestibule was measured on SPACE sequence image and the area of ELS in the cochlea and vestibule was also measured on “PPI-PEI” images using our workstation. Then, the area ratio of ELS to total fluid space was calculated. The degrees of cochlear and vestibular EH were classified as none, mild, or significant, according to the criteria reported by Nakashima et al. (20) (Nakashima's criteria). When evaluating cochlear EH, one axial slice near the modiolus was adopted. For evaluation of vestibular EH, one axial slice that displayed the vestibule to the maximum extent was adopted, but the ampulla of semicircular canal was excluded from evaluation.

Patients classified as having no EH in the cochlea showed no displacement of Reissner's membrane. Those with mild cochlear EH showed displacement of Reissner's membrane, but the area of the ELS did not exceed the area of the scala vestibuli. In patients with significant cochlear EH, the area of the ELS exceeded the area of the scala vestibuli. When the grade of EH differed between the basal and upper turns of the cochlea, the higher grade was adopted. In the vestibule, the grading was determined from the ratio of the area of ELS to that of the vestibular fluid space. Patients with no EH in the vestibule had a ratio of ≤ 1:3, those with mild EH had ratios of 1:3–1:2, and those with significant EH had a ratio >1:2. In the present study, both mild and significant EH were defined as “positive.”

##### Volumetric measurement and evaluation

Volumetric measurement of the inner ear was performed by counting voxels: voxel size = matrix size × slice thickness. First, both SPACE sequence image and “PPI-PEI” image were activated on our workstation (Virtual Place) in the present study. Second, components of the inner ear were identified on SPACE sequence image by the borderlines between the inner ear and the peripheral side of the acoustic nerve, between the end of the cochlea and the vestibule, between the three ampullae of semicircular canals (SCCs) and the vestibule, and between the distal side of the common crus and the vestibule using anatomical drawings as reported previously (19). Third, counting voxels automatically on our workstation generated volume of total fluid space. Fourth, the areas detected on SPACE sequence image were transferred to “PPI-PEI” image on our workstation: this was called “fusing.” Since MR images were obtained from one scan, all images had the same coordinates. Thus, we can perform “fusing” on workstations automatically and precisely (Figure 2). Fifth, by counting voxels of minus signals on “PPI-PEI” image, volumes of ELS were calculated. In the present study, the signal of ELS was high on FLAIR image (PPI) but almost zero on PEI image. Thus, ELS showed minus signals on “PPI-PEI” image. Finally, the ELS volume in the inner ear was measured and the ratio of the ELS area to that of the inner ear fluid space (ELS ratio) was calculated. The cochlear and vestibule areas were also measured by the same method. To evaluate the effect of ELSD, we calculated the proportion of ELS ratio (“after”/“before”). We performed these measurements three times and the average was utilized in the present study.

### Statistical Analysis

For statistical analysis, we used one-way ANOVA for multiple comparisons followed by pair-wise comparison with Tukey's *post-hoc* test and χ^2^ test or paired Student's *t*-tests for two-group comparisons. *P*-values under 0.05 were considered significant. All the data above were treated statistically with SPSS version 14.0 (Chicago, IL).

## Results

The clinical characteristics of the study cohort are summarized in Table 1.

**Table 1:** The clinical characteristics of the study cohort.

**Patient No**.	**Laterality (r/l)**	**Interval from onset to MRI (month)**	**Interval from latest vertigo to MRI (days)**
1	r	48	5
2^⋆^	l	5	0
3	r	30	30
4^⋆^	l	2	40
5	r	240	40
6	l	192	60
7	r	60	20
8	r	11	4
9	l	240	14
10	l	16	1
11	r	34	7
12	l	40	21
13	l	100	90
14	r	140	0
15	r	6	3
16	l	60	15
17	l	180	25
18	l	60	7
19	l	9	6
20	r	70	240

*The patient population consisted of four males and sixteen females, with ages ranging from 23 to 76 years (median, 61.5 years). The interval between the first vertigo attack or cochlear symptoms to MRI observation (Interval from onset to MRI) ranged from 2 to 240 months (median, 54.0 months). The interval between the latest vertigo attack to MRI observation (Interval from latest vertigo to MRI) ranged from 0 to 240 days (median, 14.5 days)*.

⋆ *Patient #2 and 4 received an MRI scan 5 months and 2 months after onset for their diagnosis, respectively. Subsequently, they suffered from recurrent episodic vertigo for 6 months and then decided to receive ELSD after treatment for 6 months. However, since the MRI had already been performed a few months prior, it was not repeated and data from the first scan were utilized*.

**Figure 3:**
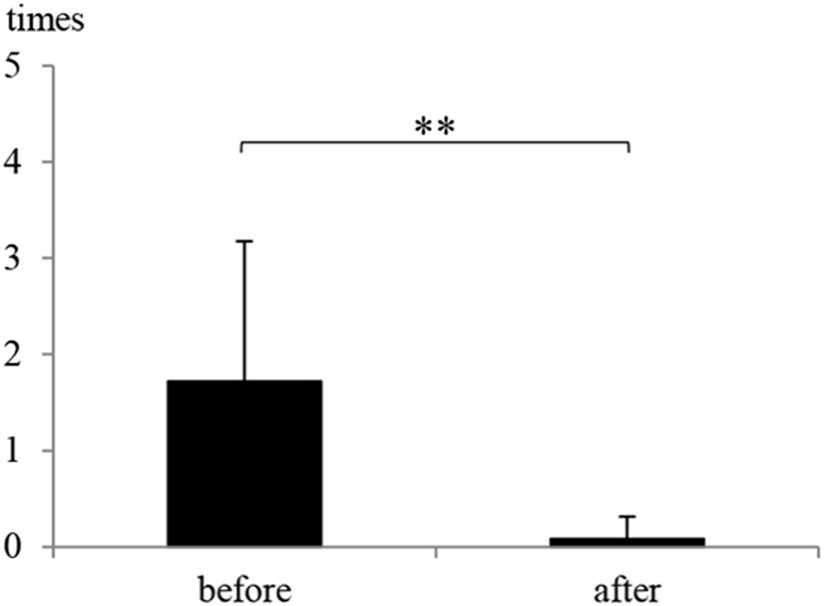
The frequency of vertigo attacks before and after endolymphatic sac drainage (ELSD). The frequency of vertigo attacks was 1.72 ± 1.46 times/month (mean ± standard deviation) before ELSD (before) and 0.08 ± 0.23 times/month after ELSD (after). The frequency decreased significantly after ELSD. **p < 0.01, paired *t*-test.

The frequency of vertigo attacks was 1.72 ± 1.46 times/month (average ± standard deviation) before and 0.08 ± 0.23 times/month after ELSD (Figure 3). The frequency decreased significantly after ELSD (*p* = 3.0 × 10^−5^, paired *t*-test). Complete control of vertigo was achieved in 80.0% (16/20) of patients (Table 2).

**Table 2:** The frequency of vertigo before and after ELSD.

**Patient No**.	**Before (times/month)**	**After (times/month)**
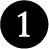	0.3	0.16
2	1	0
3	1.5	0
4	0.5	0
5	1	0
6	0.5	0
7	1	0
8	1	0
9	3	0
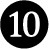	4	0.3
11	0.3	0
12	0.2	0
13	0.3	0
14	0.3	0
15	3	0
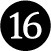	2.5	1
17	4	0
18	4	0
19	2	0.2
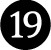	4	0

*The frequency of vertigo before ELSD (“before”) was calculated based on the number of vertigo attacks during the 6 months before ELSD. Frequency after ELSD (“after”) was calculated based on the number of vertigo attacks during the 6-month period between 18 and 24 months after ELSD. After ELSD, 80% (16/20) of patients showed relief from vertigo. Patients with circled numbers and the underlined values (#1, 10, 16, and 19) still suffered from vertigo after ELSD*.

### Functional Measurement

**Figure 4:**
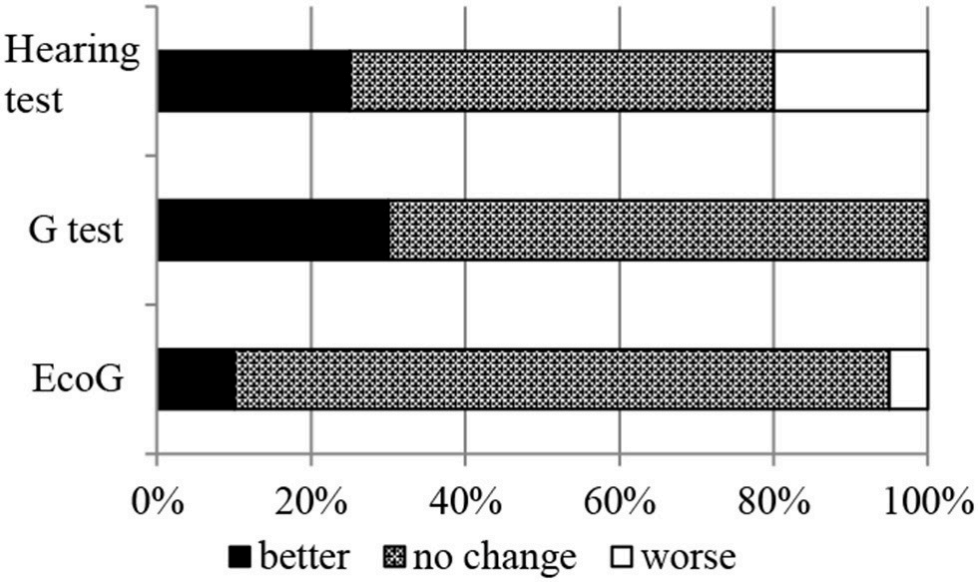
Changes in the result of hearing tests, glycerol tests (G-test), and electrocochleography (EcoG). After ELSD, 25.0% of patients (5/20) showed hearing improvement, 30.0% (6/20) showed negative conversion of G-test findings, and 10.0% (2/20) showed negative conversion of EcoG findings. There was no relationship between complete control and the results of the hearing test, G-test, and EcoG.

Hearing improvement was observed in 25.0% of patients (5/20), negative conversion of G-test results in 30.0% (6/20), and negative conversion of EcoG findings in 10.0% (2/20) (Figure 4). There was no relationship between complete control and the results of the hearing test, G-test, and EcoG.

### MRI Assessments

**Figure 5:**
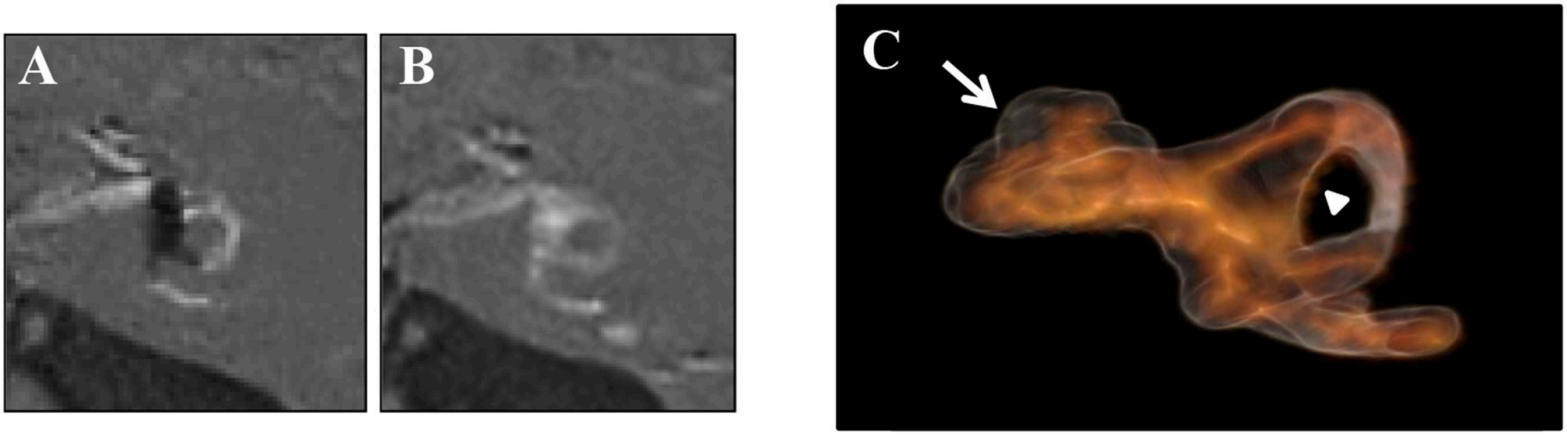
Changes in MRI images after ELSD. **(A)** Images of “PPI-PEI” before ELSD. **(B)** Images of “PPI-PEI” after ELSD. **(C)** Reconstructed 3D images of inner ear. There are non-enhanced areas with Gd, suggesting the existence of EH, in the cochlea, vestibule, and semicircular canals (SCCs) of the image obtained before ELSD **(A)**. In contrast, non-enhanced areas in the vestibule and SCCs disappeared after ELSD **(B)**. On reconstructed 3D image, light-browned area represents the perilymphatic space and the black area represents ELS **(C)**. White arrow indicates ELS (=EH) in the cochlea and arrow head indicates ELS (=EH) in the vestibule.

Figures 5A,B showed typical changes in MRI images after ELSD. There are non-enhanced areas with Gd, indicating the existence of EH, in the cochlea, vestibule, and SCCs of the image obtained before ELSD (Figure 5A). In contrast, non-enhanced areas in the vestibule and SCCs disappeared after ELSD (Figure 5B). Figure 5C showed the reconstructed 3D image of the perilymphatic space and ELS.

#### Conventional Two-Dimensional Evaluation

**Figure 6:**
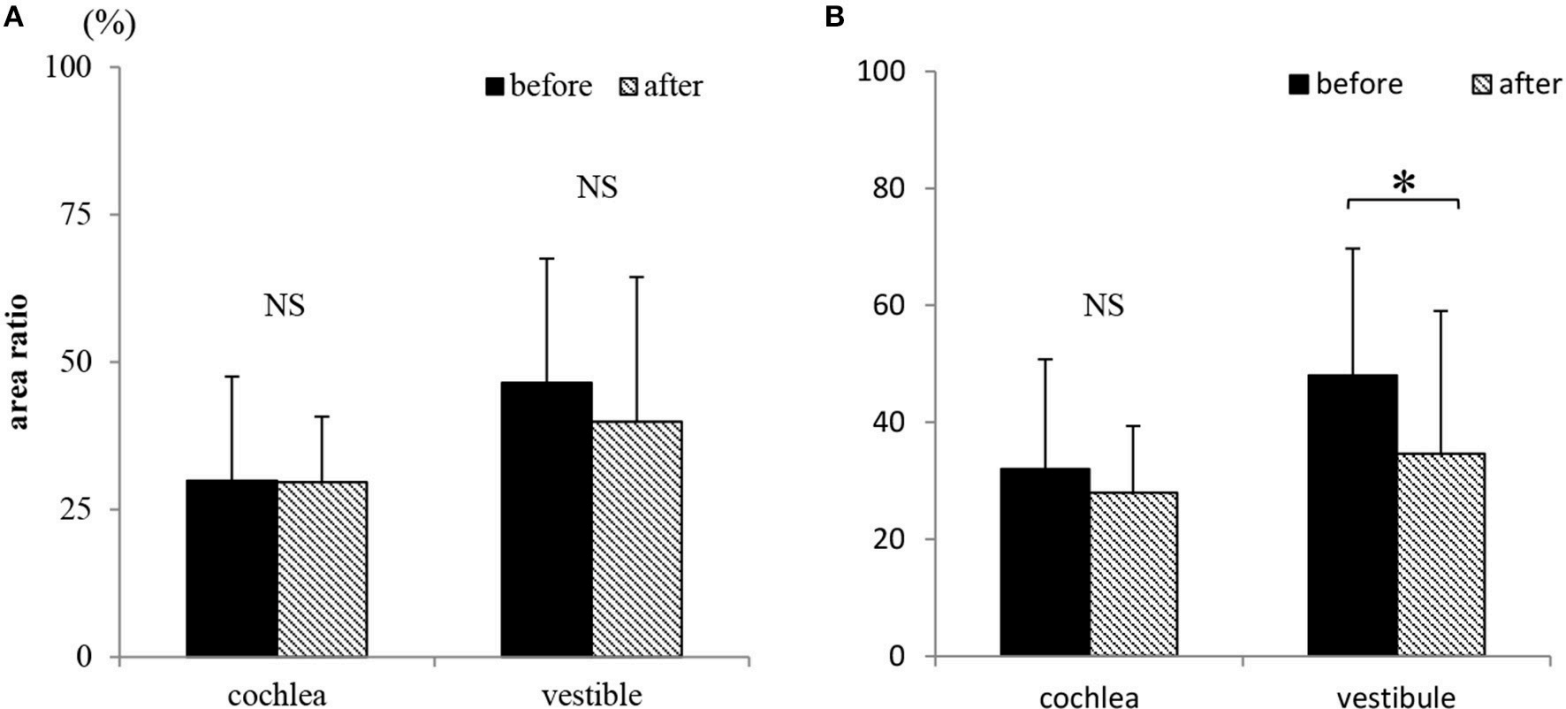
Changes in the existence of endolymphatic hydrops (EH) on conventional two-dimensional evaluation. **(A)** The area ratio of EH in cochlea and vestibule before and after ELSD among all 20 patients. **(B)** The area ratio of EH in cochlea and vestibule before and after ELSD among patients under complete of vertigo after ELSD Among all patients, the area ratio of EH to total fluid space was 29.9 ± 17.6% (average ± standard deviation) in the cochlea and 46.5 ± 21.1% in the vestibule before ELSD. After ELSD, the area ratio was 29.6 ± 11.1% in the cochlea and 39.9 ± 24.6% in the vestibule **(A)**. There was no difference of area ratio between the before and after groups. Among patients under complete control of vertigo, the area ratio was 32.0 ± 18.8% in the cochlea and 48.0 ± 21.7% in the vestibule before ELSD. After ELSD, the area ratio was 27.9 ± 11.4% in cochlea and 34.6 ± 24.4% in the vestibule **(B)**. There was no difference of the area ratio in the cochlea between before and after ELSD but in the vestibule the area ratio significantly decreased after surgery. *p < 0.05, paired *t*-test; NS, not significant, paired *t*-test.

Figure 6 and Table 3 show the area ratio of EH to total fluid space (area ratio) in the cochlea and vestibule. There was no significant difference in area ratio between before and after ELSD among all patients (Figure 6A: NS, paired *t*-test). Among patients with complete vertigo control, the area ratio of the cochlea tended to decrease after ELSD but there were no significant differences between before ELSD and after. In contrast, the area ratio of the vestibule decreased significantly after ELSD (Figure 6B: *p* = 0.02, paired *t*-test). The incident ratio of EH [by Nakashima's criteria (20)] is also described in Table 3. There was no relationship between complete control of vertigo and the expression of EH (Table 3).

**Table 3:** Changes in the existence of EH after ELSD.

**Patient No**.	**Cochlea**				**Vestibule**			
	**Before**		**After**		**Before**		**After**	
	**Area ratio (%)**	**EH**	**Area ratio (%)**	**EH**	**Area ratio (%)**	**EH**	**Area ratio (%)**	**EH**
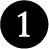	26.9	Positive	25.9	Positive	59.1	Positive	53.7	Positive
2	36.4	Positive	27.3	Positive	27.3	Negative	28.0	Negative
3	44.0	Positive	26.1	Positive	67.8	Positive	53.3	Positive
4	71.4	Positive	12.5	Negative	37.5	Positive	7.4	Negative
5	20.0	Negative	36.0	Positive	23.8	Negative	25.9	Negative
6	60.0	Positive	46.2	Positive	44.4	Positive	0	Negative
7	28.6	Positive	36.8	Positive	25.9	Negative	33.3	Positive
8	17.6	Negative	13.0	Negative	23.8	Negative	8.7	Negative
9	26.3	Positive	22.7	Negative	66.7	Positive	14.2	Negative
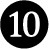	22.2	Negative	38.4	Positive	36.8	Positive	55.0	Positive
11	10.7	Negative	25.8	Positive	56.0	Positive	42.4	Positive
12	0	Negative	20.0	Negative	26.3	Negative	31.5	Negative
13	7.7	Negative	13.6	Negative	24.1	Negative	11.5	Negative
14	42.9	Positive	29.2	Positive	81.8	Positive	61.5	Positive
15	30.8	Positive	50.0	Positive	48.0	Positive	83.3	Positive
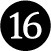	28.6	Positive	44.4	Positive	14.3	Negative	75.0	Positive
17	41.7	Positive	36.8	Positive	73.3	Positive	39.1	Positive
18	30.8	Positive	17.4	Negative	81.0	Positive	37.0	Positive
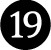	8.3	Negative	36.8	Positive	51.7	Positive	60.0	Positive
20	42.9	Positive	33.3	Positive	60.0	Positive	76.2	Positive

*The area of total fluid space and ELS in the cochlea and vestibule were measured and the area ratio of ELS to total fluid space was calculated (area ratio). EH was also evaluated by Nakashima's criteria. Both mild and significant EH were defined as “positive.” Before ELSD, the area ratio was 29.9 ± 17.6% (average ± standard deviation) in the cochlea and 46.5 ± 21.1% in the vestibule. After ELSD, the area ratio was 29.6 ± 11.1% in the cochlea and 39.9 ± 24.6% in the vestibule. There was no difference of area ratio between before and after (NS, not significant; paired t-test). The incident ratio of EH (positive) was 65.0% (13/20) in the cochlea, 65.0% (13/20) in the vestibule, and 80.0% (16/20) in total. In contrast, after ELSD, the incident ratio was 70.0% (14/20) in the cochlea, 60.0% (12/20) in the vestibule, and 75.0% (15/20) in total. There were no significant differences between the “before” and “after” values (NS, χ^2^-test). Patients with circled numbers still suffered from vertigo after ELSD, and the others were free from vertigo*.

#### Volumetric Measurement and Evaluation

**Figure 7:**
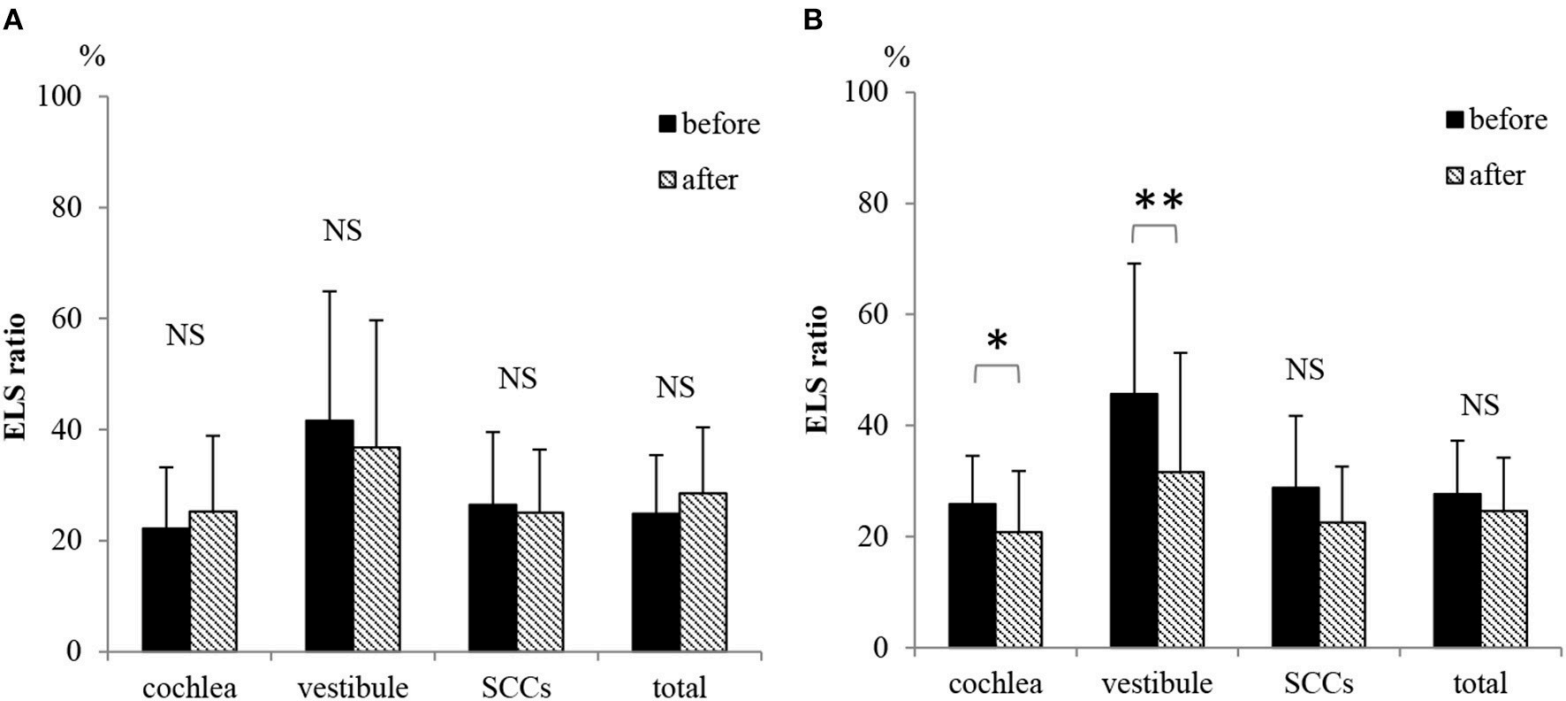
Changes in the ELS ratio after ELSD. **(A)** ELS ratio in the cochlea, vestibule, SCCs, and whole inner ear (total) before and after ELSD among all 20 patients. **(B)** ELS ratio in the cochlea, vestibule, SCCs, and total before and after ELSD among patients under complete control of vertigo after ELSD. The average of ELS ratio before ELSD among all 20 patients was 22.2 in the cochlea, 41.6 in the vestibule, 26.4 in SCCs, and 24.8 in total. The average ELS ratio after ELSD among all patients was 24.6 in the cochlea, 35.7 in the vestibule, 25.0 in SCCs, and 28.0 in total **(A)**. There were no differences in the ELS ratio before and after ELSD (paired *t*-test). The average ELS ratio before ELSD among patients showing complete control of vertigo was 25.9 in the cochlea, 45.7 in the vestibule, 28.8 in SCCs, and 23.7 in total. In contrast, the average ELS ratio after ELSD was 19.7 in the cochlea, 29.9 in the vestibule, 22.3 in SCCs, and 23.7 in total **(B)**. There were significant differences between the ELS ratios before and after ELSD in the cochlea and in the vestibule (paired *t*-test). On the other hand, the ELS ratio tended to decrease in SCCs and in total but showed no significant differences. *p < 0.05, paired *t*-test, **p < 0.01, paired *t*-test; NS, not significant, paired *t*-test.

**Figure 8:**
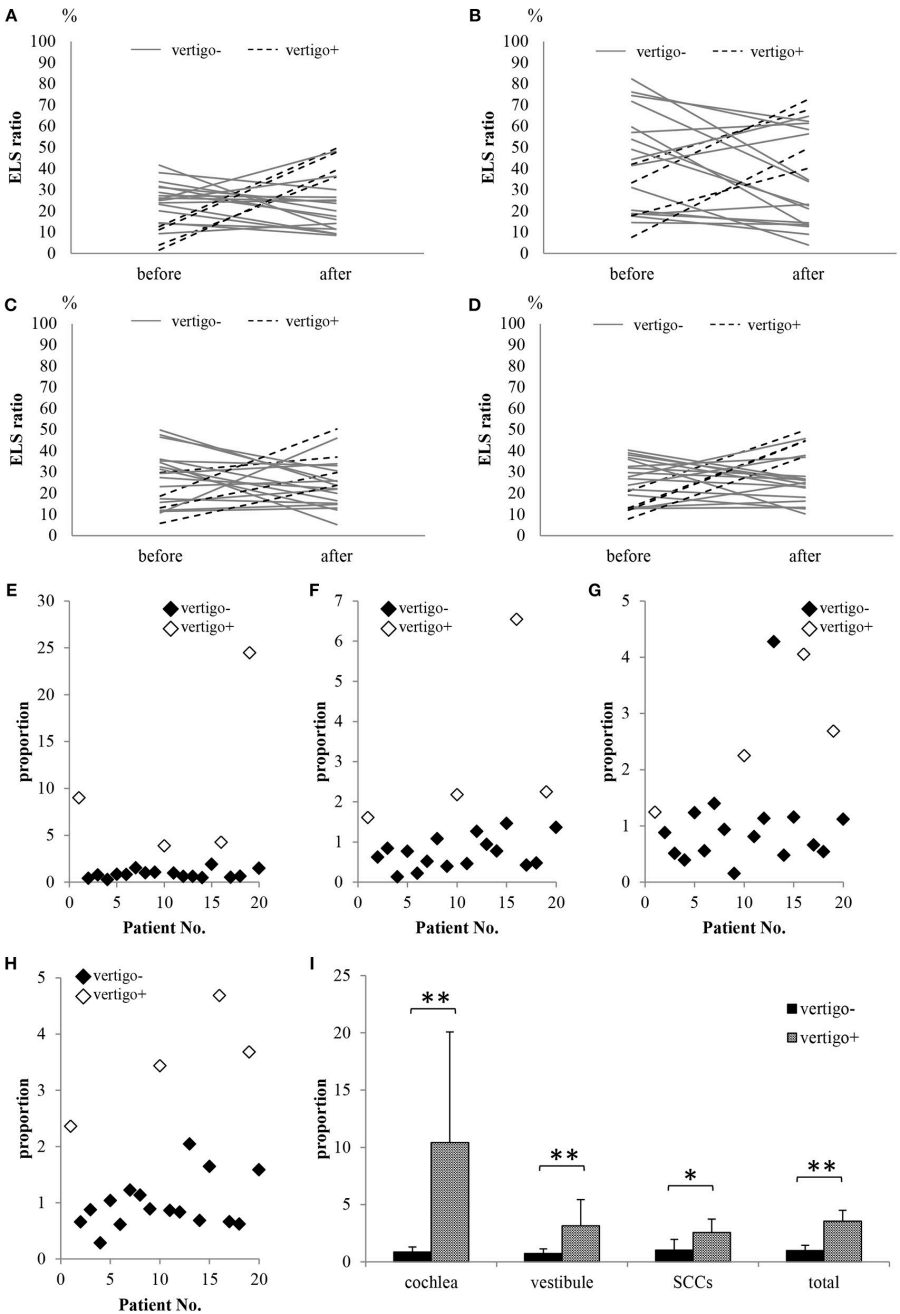
Comparison of the ELS ratio in patients showing complete control of vertigo with patients showing vertigo after ELSD. (**A)** ELS ratio in the cochlea. **(B)** ELS ratio in the vestibule. **(C)** ELS ratio in SCCs. **(D)** ELS ratio in the cochlea, vestibule, and/or SCCs (total). **(E)** Proportion of ELS ratio in the cochlea after ELSD compared to that before ELSD. **(F)** Proportion of ELS ratio in the vestibule after ELSD compared to that before ELSD. **(G)** Proportion of ELS ratio in the SCCs after ELSD compared to that before ELSD. **(H)** Proportion of ELS ratio in total after ELSD compared to that before ELSD. **(I)** Comparison of proportion in patients showing complete control of vertigo (vertigo–) with patients showing vertigo after ELSD (vertigo+). Almost all vertigo– patients showed decreased ELS ratios for the cochlea, vestibule, SCCs, and total after ELSD. In contrast, all vertigo+ patients showed increased ELS ratios after ELSD **(A–D)**. The proportions in ELS ratios in the vertigo– patients were significantly lower than those in vertigo+ patients in the cochlea, vestibule, SCCs, and total **(E–I)**. Gray lines: vertigo– patients; dotted lines: vertigo+ patients; black diamonds: vertigo– patients; white diamonds: vertigo+ patients. *p < 0.05, one-way ANOVA, Tukey test, **p < 0.01, one-way ANOVA, Tukey test.

Figure 7A shows the ELS ratios in the cochlea, vestibule, SCCs, and whole inner ear (total) among all 20 patients. There were no differences in the ELS ratios before and after ELSD (total: *p* = 0.18, cochlea: *p* = 0.25, vestibule: *p* = 0.22, and SCCs: *p* = 0.37, paired *t*-test). Figure 7B shows the ELS ratio among patients with complete control of vertigo (vertigo–). A comparison of all patients showed that the ELS ratio in the cochlea and vestibule after ELSD were significantly smaller than before ELSD (cochlea: *p* = 0.03, vestibule: *p* = 0.008, paired *t*-test). The ELS ratio in SCCs and in total tended to decrease after ELSD but showed no significant differences between before and after ELSD (*p* = 0.06, paired *t*-test). Figures 8A–D shows the changes in the ELS ratio after ELSD in each patient. Almost all patients with complete control of vertigo (vertigo–, gray lines) showed lower ELS ratios after ELSD. In contrast, patients still suffering from vertigo (vertigo+, dotted lines) showed increased ELS ratios after ELSD. As seen in Figures 8E–I, the proportion of the ELS ratio of vertigo– was significantly low in the cochlea, vestibule, SCCs, and in total (cochlea, vestibule, and total: *p* < 0.01, one-way ANOVA, Tukey test, SCCs: *p* < 0.05, one-way ANOVA, Tukey test).

## Discussion

In the present study, we first reported the volumetric change of ELS after ELSD, suggesting the relationship between the reduction of vertigo attack and decreased volume of ELS.

In 1938, Yamakawa in Japan and Hallpike in England reported the pathology of MD to involve EH (1, 2). Since then, treatments for MD have focused on reducing EH. In 1927 Portmann introduced ELSD as an effective surgery to control EH (8). In 2008, Kitahara et al. modified the surgery with steroid instillation into the opened sac and reported good results for vertigo control and hearing (14). In contrast, some groups reported that ELSD had debatable efficacy based on the results of placebo-control studies (21, 22). Most studies on ELSD assessed its effects on reducing the frequency of vertigo since the EH could not be detected in live patients before Nakashima et al. introduced an approach to visualize the EH using 3T MRI in 2007.

Some reports have discussed the changes in EH after ELSD using 3T MRI and conventional two-dimensional evaluations (9, 23). They reported that suppression of vertigo was not always the result of a reduction in EH and that there was no relationship between suppression of vertigo and reduction of EH. In the present study, we also observed changes in EH using conventional two-dimensional evaluation (Nakashima's criteria) and could not find a relationship between vertigo suppression and EH. Then, we attempted volumetric measurement of the ELS as reported by our group previously (19). In the present study, 80.0% of patients experienced relief from vertigo after ELSD (vertigo–). However, the remaining 20.0% still suffered from vertigo (vertigo+). The ELS ratio of vertigo– patients had significantly decreased after ELSD, while that of vertigo+ patients did not show a significant change. We previously reported the volume of ELS and ELS ratio of controls that had never experienced vertigo/dizziness and never suffered ear disease; the volume of ELS in controls was 10.3 ± 6.9 μl (average ± standard deviation) in the cochlea and 11.5 ± 7.1 μl in the vestibule. The ELS ratio in the cochlea was 8.8 ± 5.3 and that in the vestibule was 16.2 ± 9.0 (19). Compared with the controls, the ELS ratio of vertigo– patients was still higher. Chung et al. conducted a temporal bone study and reported that EH persisted in all patients that received ELSD, even though the vertigo was well-controlled (24). Thus, ELSD can reduce EH, but it cannot achieve normalization. These findings suggest that if EH can be reduced by ELSD, the patient's vertigo could be controlled. In the present study, 20.0% of patients still experienced vertigo and showed an increased ELS ratio. The opened sac might have closed over 2 years and could not reduce EH in these patients.

Gürkov et al. reported betahistine treatment in six patients with MD did not change EH, although their symptoms improved significantly (25). Fiorino et al. also reported intra-tympanic gentamicin therapy did not reduce EH even though vertigo attacks were disappeared (26). They adopted two-dimensional evaluations and reported no remarkable effect on EH despite suppression of vertigo. In the present study, we first reported the changes in EH using volumetric measurement and revealed significant changes in EH in the vertigo– group. Assessment of the EH using this method would allow identification of changes in and the effects on EH after treatment (i.e., isosorbide, steroids, Meniett) and help clinicians decide whether to continue the treatment.

The limitation of the present study is the small number of patients. Because ELSD can often reduce the frequency of vertigo, we experienced difficulty in collecting data on patients showing vertigo even after ELSD. Nevertheless, by comparing the proportions of the ELS ratio of “after treatment” and “before treatment” groups, we could understand the extent to which EH could be reduced to control vertigo. This could facilitate future progress in the field of treatment for MD.

## Author Contributions

TI, TK, KK, NT, and TY designed the research. TI, HI, TM, TS, and SM performed the research. TI analyzed the data and wrote the paper.

### Conflict of Interest Statement

The authors declare that the research was conducted in the absence of any commercial or financial relationships that could be construed as a potential conflict of interest.
